# Building a recruitment database for asthma trials: a conceptual framework for the creation of the UK Database of Asthma Research Volunteers

**DOI:** 10.1186/s13063-016-1381-6

**Published:** 2016-05-26

**Authors:** Bright I. Nwaru, Ireneous N. Soyiri, Colin R. Simpson, Chris Griffiths, Aziz Sheikh

**Affiliations:** Asthma UK Centre for Applied Research, Centre for Medical Informatics, Usher Institute of Population Health Sciences and Informatics, Medical School, The University of Edinburgh, Teviot Place, Doorway 3, Edinburgh, EH8 9AG UK; School of Health Sciences, University of Tampere, Tampere, Finland; Centre for Primary Care and Public Health, Blizard Institute, Queen Mary University of London, London, UK

**Keywords:** Asthma, Clinical trials, Electronic health records, Data linkage, Recruitment database

## Abstract

**Background:**

Randomised clinical trials are the ‘gold standard’ for evaluating the effectiveness of healthcare interventions. However, successful recruitment of participants remains a key challenge for many trialists. In this paper, we present a conceptual framework for creating a digital, population-based database for the recruitment of asthma patients into future asthma trials in the UK. Having set up the database, the goal is to then make it available to support investigators planning asthma clinical trials.

**Methods:**

The UK Database of Asthma Research Volunteers will comprise a web-based front-end that interactively allows participant registration, and a back-end that houses the database containing participants’ key relevant data. The database will be hosted and maintained at a secure server at the Asthma UK Centre for Applied Research based at The University of Edinburgh. Using a range of invitation strategies, key demographic and clinical data will be collected from those pre-consenting to consider participation in clinical trials. These data will, with consent, in due course, be linkable to other healthcare, social, economic, and genetic datasets. To use the database, asthma investigators will send their eligibility criteria for participant recruitment; eligible participants will then be informed about the new trial and asked if they wish to participate. A steering committee will oversee the running of the database, including approval of usage access. Novel communication strategies will be utilised to engage participants who are recruited into the database in order to avoid attrition as a result of waiting time to participation in a suitable trial, and to minimise the risk of their being approached when already enrolled in a trial.

**Results:**

The value of this database will be whether it proves useful and usable to researchers in facilitating recruitment into clinical trials on asthma and whether patient privacy and data security are protected in meeting this aim.

**Conclusions:**

Successful recruitment is fundamental to the success of a clinical trial. The UK Database of Asthma Research Volunteers, the first of its kind in the context of asthma, presents a novel approach to overcoming recruitment barriers and will facilitate the catalysing of important clinical trials on asthma in the UK.

**Electronic supplementary material:**

The online version of this article (doi:10.1186/s13063-016-1381-6) contains supplementary material, which is available to authorized users.

## Background

Randomised clinical trials are the ‘gold standard’ for evaluating the effectiveness of interventions in reaching medical and policy decisions [1, 2]. Often clinical trials run into difficulties, with slow or inadequate recruitment of participants being a particularly common reason for trials failing to deliver [3–9]. In the UK, only 55 % of trials funded by the National Institute of Health Research Health Technology Assessment (NIHR HTA) and the Medical Research Council achieved their original recruitment target on time, whilst 45 % received an extension of time to recruit [4, 6]. This trend shows no sign of improvement over time [4].

Recruitment is an important measure by which the overall success of a trial may be gauged [6, 9]. Poor recruitment gives rise to underpowered studies or leads to extension of overall time lines for the completion of trials, often incurring additional costs and compromising the relevance of findings [7, 9]. Strategies to improve recruitment into trials include telephone reminders, opt-out procedures, and open designs, but their impact on enhanced recruitment appears to be marginal [5, 8, 9].

**Fig. 1:**
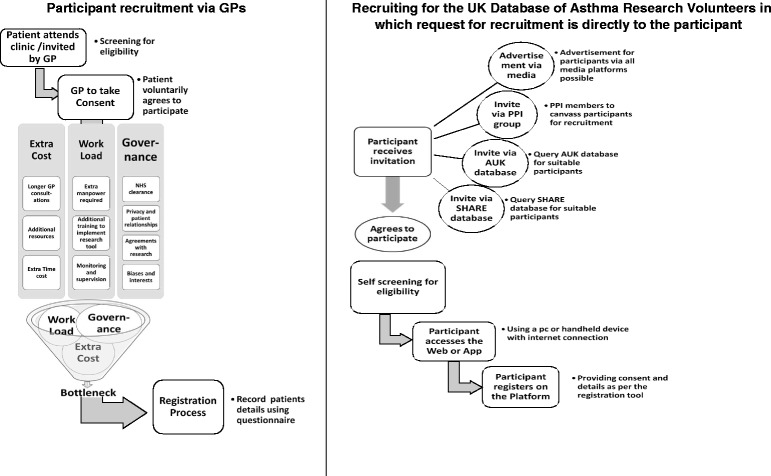
A schematic contrast of general practitioner (GP) and open recruitment for potential clinical trials showing the underlying challenges

There is growing interest in the potential of routine healthcare data, particularly electronic health records (EHRs), to enhance the efficiency of conducting clinical trials [10–14]. The feasibility and success of utilising stand-alone or linked routine datasets in conducting trials has been evaluated, and clearly evidence points in the direction of improved recruitment and conduct of clinical trials [10–14]. With respect to participant recruitment, EHRs have been used for point-of-care enrolment and randomisation, with clinicians employing opportunistic patient recruitment strategies during consultation or using information systems such as flagging or care-scheduling pop-ups [13, 15]. Whilst most clinicians are, in principle, generally in favour of becoming involved in recruiting patients into trials, actual involvement can be complex and time-consuming for often already over-stretched frontline clinical staff [4, 6, 8, 16, 17]. Time constraints, impact on clinician-patient relationship, and added workload to already busy schedules constitute other considerations that may hinder clinicians’ involvement in patient recruitment (Fig. 1) [4, 6, 8, 16, 17]. This results in patients seldom being invited to participate, even though research suggests that many patients are willing to be recruited into trials [8, 16]. There is, therefore, a need to find novel approaches that do not add to the workload of clinician gatekeepers by approaching patients directly with a view to offering them the opportunity to participate in clinical trials [16].

In this paper, we present a conceptual framework for creating a database for the recruitment of volunteers who are willing and eligible to participate in future asthma clinical trials in the UK. The UK ranks among the countries with the highest prevalence, healthcare utilisation and mortality from asthma [18, 19]; therefore, asthma serves as an appropriate exemplar disease condition for creating this database. Successful development of this initiative in this common clinical area could pave the way for similar developments in other chronic disease areas. The overall goal is to establish a digital population-based database of asthma patients across all age groups who pre-consent to be contacted to take part in future asthma clinical trials and give their consent for their EHRs to, in due course, be scrutinised and cross-linked to other health, genetic, social, and economic datasets in order to determine eligibility to participate and tailor invites accordingly. Having set up the database, the goal is to then make it available to investigators planning a range of asthma clinical trials from across the UK. We also present selected analogous existing clinical research recruitment databases (Additional file 1) that serve as ‘learning experiments’ for highlighting the feasibility, features, benefits, and challenges of the proposed database [20–23]. Finally, we discuss the likely added values of the database to research, practice, and policy as we aim to increase the efficiency with which clinical trials on asthma are undertaken in the UK.

## Methods

### Database design and set-up

The design and set-up of the database will closely follow the approaches used to establish the generalist National Health Service Scotland (NHS Scotland) Scottish Health Research Register (SHARE) which targets all people in Scotland for recruitment [16]. There are, however, some modifications with regards to the management of the recruitment process for trials being undertaken within the database and, more fundamentally, the UK-wide focus of our initiative. After careful consideration of the structural set-up and applicable recruitment management processes of similar existing databases (Additional file 1), we decided to follow the Duke Clinical Research Unit (DCRU) model [22], in which the database administrators are involved in all stages of the recruitment process for each study undertaken within the database. Whereas SHARE allows investigators using the database direct contact with potential participants, the DCRU team directly undertakes the recruitment process on behalf of each study and manages the process until the end of the study. This, we believe, ensures the opportunity for careful management of the recruitment process, minimises the risk of exploitation of participants’ privacy from investigators, and consequently will provide greater confidence from participants and ensure their continuous involvement in the database.

**Fig. 2:**
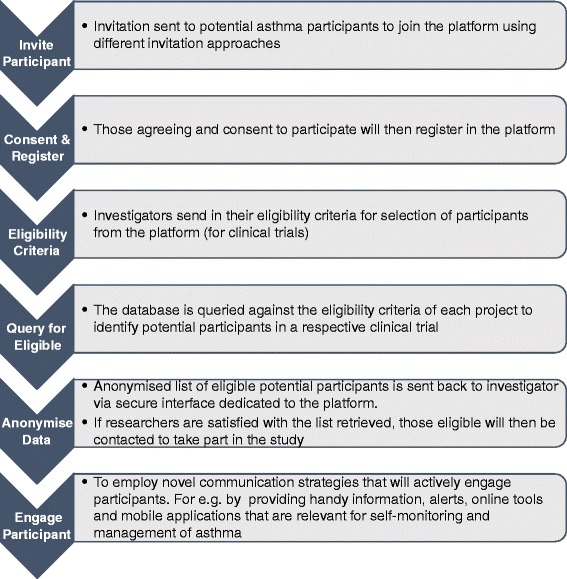
Strategies to populate and use the UK Database of Asthma Research Volunteers

The database will comprise front-end and back-end components. The front-end refers to the interface where users interact with a computer or handheld electronic device through which they are able to browse or navigate using tools like icons and dropdown menus. On the other hand, the back-end is the underlying database structure and the technology that houses databases, web applications, and helps to power the components that enable the user-face (front-end) of the website to perform optimally. After setting up the database, the fundamental strategies to populating and utilising the database will involve seven integrated stages as outlined and explained below and illustrated in Fig. 2:

1. Invitation sent to potential asthma participants to join the database using different invitation approaches: all those invited are asked to complete a short questionnaire asking for some demographic data, such as age, sex, and place of residence
2. Those who agree and consent to participate will then register in the database
3. Information on volunteers’ asthma status and current use of asthma medications will be requested
4. Asthma research investigators send in their eligibility criteria for selection of participants from the database to take part in planned clinical trials
5. The database is queried against the eligibility criteria of each project to identify potential participants in respective clinical trials
6. The list of eligible potential participants (containing basic demographics but without any identifiable participant information) is sent back to researchers through a secure interface that will be dedicated to the database. If researchers are satisfied with the list, those who are eligible will then be invited to take part in the study
7. During the life of a specific trial and continuously for the database, we will employ novel communication strategies to continue to engage participants already recruited into the database

Investigators will have the opportunity to undertake emerging clinical design strategies using the database if needed. For example, using the stepped-wedge design, general practitioner (GP) practices can serve as useful clusters for randomisation. Other possible designs that can be implemented include n-of-1 trials (which can be enhanced through wearable data that can be collected and directly populated in the platform’s secure database for further processing) and adaptive clinical trials, which can provide an opportunity to implement relevant adaptations to the trial and statistical procedures without major contamination of the planned trial process given that the population recruited into the platform should remain stable over time. In addition, to advancing multi- and cross-disciplinary research, the database will provide an infrastructure to link data from the database to other health, social, economic, and genetic datasets on an ongoing basis.

**Fig. 3:**
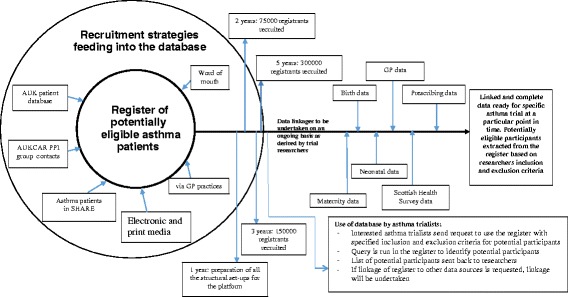
Diagrammatical description of the UK Database of Asthma Research Volunteers. Platform for Recruitment and Conduct of Clinical Research on Asthma

Diagrammatically, the set-up, recruitment approaches, and milestones of the database are shown in Fig. 3. The population in the database will include people of all ages. We will devote the first year to building the relevant structures for full launch of the database, including: piloting a beta version of the database, securing further funding; establishing ethical and governance oversight committees; setting up remote and physical study sites; creating integrated recruitment approaches; establishing collaborations across the UK; and identifying candidate asthma projects that can first use the database. In order to maintain a continuous retention of participants within the database, our aim is to start recruitment of participants into small-scale clinical trials as soon as possible after sufficient (i.e. *n* >1000) participant enrolment into the database. Participants are likely to withdraw their participation from the database if it takes too long to initiate their participation in a study from when they enrolled in the database. Therefore, during the first year of preparatory work for setting up the database, we will concurrently identify potential asthma clinical trials across the UK that are at the planning stages and liaise with the investigators on utilising the database for their projects once launched. In this way, recruited registrants who are eligible for participation in a particular trial will have the opportunity to be enrolled into a study within a short time from registration. From the second year, we aim to achieve recruitment of at least 75,000 registrants per year to a final 300,000 during the first 5 years.

### Recruitment approaches

Recruitment strategies of potential participants will be carried out via:

1. Asthma UK – we are working in close partnership with Asthma UK, the national patient charity and support organisation, to recruit participants through their existing databases. Asthma UK will play a major role in supporting our recruitment campaign
2. Members of our Patient and Public Involvement (PPI) Group will help in canvassing participants for recruitment through handing out of flyers and word of mouth
3. A snowball sampling approach will be used to reach out to participants by asking those already enrolled in the database to help invite their network of contacts
4. Approaching people who are already participating in asthma studies, and people with asthma who are participating in non-asthma studies
5. Capitalising on the opportunities offered by both the electronic and print media, including strategic advertisements on local television and radio stations, the Internet, and local newspapers in reaching out to potential participants
6. SHARE – we will search the SHARE database in order to identify potentially eligible asthma patients who are already taking part in SHARE and invite them to participate. We will seek to develop a reciprocal arrangement whereby database volunteers would be asked to participate in SHARE and vice versa
7. GP practices – we will liaise with GP practices to invite participants through personalised direct mail endorsed by the patients’ GP. In addition, we will enclose invitation letters with outpatient appointment letters to eligible participants

Those who consent to enrol into the database will complete an enrolment form that asks for some basic demographic information and information that assesses their asthma status (Additional file 2).

### Project time lines and milestones

The project time lines are shown in Fig. 3 to show the anticipated milestones within the first 5 years.

### Cross-data linkages and data management

Given the possibilities that are now available in the UK to undertake deterministic cross-data linkages using the unique identifiers, Community Health Index (CHI) number in Scotland and NHS numbers in England, Wales and Northern Ireland, having set up the database, we aim, in due course, to link data from the database on an ongoing basis to other routinely-collected health, genetic, economic, and social datasets for participants who consent to allow us to undertake data linkages of their data. These data linkages will provide the opportunity for recruiting participants into trials that will be investigating both clinical and non-clinical asthma outcomes; they will also enhance the possibility of undertaking long-term follow-up of clinical trials to be undertaken using the database.

The back-end of the database will collate and populate the relevant data obtained from enrolled participants. The Structured Query Language (SQL) routines (with enabled features to easily convert to other database formats) will be used as the default programme to manage participants’ data. The collected data will automatically be encrypted and routed to the project database systems and hosted via a secure portal at The University of Edinburgh. These data will be stored on secure encrypted servers with password-secured access restricted to designated database administrators. The database management administrators will be responsible for all issues related to data coding, security, access and audit. Administrators will ensure compliance with Data Protection Act Principles [24]. The database administrator will work closely with the database investigators and the steering committee in developing all functions required for data management. The register will be routinely monitored for changes in population dynamics. Standard data quality checks will be performed and reported on an ongoing basis.

### Ethics, governance, and data security

NHS Research Ethics Committee approval is not required for setting up a research volunteer database, but we will apply for ethics approval when we require further patient information, need to search GP records and undertake data linkages.

A steering committee is being set up, involving leading project investigators and representatives of Asthma UK, government and patient and public groups. The committee’s primary tasks will be to oversee the running of the database, including approval of access to use the database, review of data-gathering tools/approaches used within the database, approval of data requests, provision of guidance on dealing with privacy and security issues arising from using the database, and provision of general advice on the day-to-day running of the database as these arise. The committee will also guide and ensure that data management procedures meet and align with the project’s strategic interest in asthma clinical trials.

### Collaborators

We are collaborating with Asthma UK and will seek additional collaborators as and when required.

### The role of the patient groups

Within the Asthma UK Centre for Applied Research, a PPI group is involved in asthma projects to ensure that the perspectives of patients and families affected with asthma are clearly integrated. We have, therefore, engaged the PPI group throughout the processes of developing the database. At each stage of development; for example, when developing the database prototype, we sought and integrated the PPI group feedback. We will continue to work with the PPI group as we progress with the development of the database.

### Project status

Most preliminary background work for establishing the database has been completed. We have now liaised with investigators of similar databases (Additional file 1), with the view of understanding their experiences that we hope will be integrated in developing the frameworks for the current database. Building on the understanding gained from these discussions, we have now contracted an IT company to build the database, and work has progressed well on this. The ultimate plan is to ready the database by the end of August 2016, test its performance using our PPI group for the next 1-2 months, and then prepare for launch in autumn of 2016.

## Results

The value of this database will primarily be assessed by whether it proves useful and usable to researchers in facilitating recruitment into clinical trials on asthma and whether patient privacy and data security are protected in meeting this aim. A long-term ambition of electronic health record systems is to facilitate patient access to data. This is important to ensure record accuracy and completeness, whilst being able to record the provenance of data. Our long-term ambition for this database is, therefore, to facilitate patient access to data, which is a major UK policy; we plan to implement this component in the database in due course.

## Discussion

We have outlined the conceptual framework for creating a digital population-based database for the recruitment of participants who are eligible for, and consent to take part in, future UK asthma clinical trials. To the best of our knowledge, this is the first digital population-based recruitment database specifically devoted for the conduct of clinical trials on asthma. Although other analogous databases exist in other parts of the world, as already highlighted in Additional file 1, most of these, apart from the Scottish Diabetes Research Network, are generalist databases, i.e. they do not have any particular disease focus. Although there is no empirical evidence comparing the potentials of disease-specific and generalist recruitment databases, our discussions with asthma patient groups indicate that potential participants appear open to databases that are tailored to their specific disease conditions. An asthma recruitment portal dedicated for the conduct of clinical trials on asthma has immense future potential for improving health of people affected with asthma. With a recruitment target of 300,000 participants in the first 5 years of setting up the database through a range of recruitment avenues, we will be in a position to undertake suitably powered clinical trials to evaluate key questions on the management of asthma. With the capacity to undertake cross-data linkages, the database will consequently provide potentials for addressing wide-ranging research questions on asthma, including questions of intervention effects on different asthma phenotypes, patient-reported asthma outcomes, and an opportunity towards key targets of implementing stratified healthcare in asthma.

Whilst it is possible that those with more labile and poorly controlled asthma may more likely register to the database, we think that such selective recruitment will potentially impact on the external validity (or generalisability) of the trials undertaken using the database, and not on the internal validity. In developing this database, our approach has been facilitated through extensive discussions with asthma patients, of varying degrees of asthma severity, who have supported this work and expressed an interest in participating for largely altruistic reasons. Whilst we acknowledge that risk of selection potentially remains, we believe that it is likely to be diminished over and above the current status quo.

With the set-up of this database, we have the prospect of substantially transforming the landscape of conducting clinical trials on asthma in the UK. We hope that the time taken for recruitment of participants into clinical trials will substantially reduce, with the implication that clinical trials on asthma can be undertaken in a more timely and cost-efficient manner. We hope that such a resource as the current database will contribute to attracting the interest of international asthma investigators to the UK in order to undertake cutting-edge clinical trials on asthma. Finally, we believe that the database will also provide a catalyst to help start cross-sectoral and cross-national collaborations, which will provide key outputs to influence policy, practice, and research targets for the improved management of asthma across all age groups.

## Conclusion

Successful recruitment is a key determinant of the ultimate success of any clinical trial. This database, the first of its kind in the context of asthma, presents a novel approach to overcoming current recruitment barriers in conducting clinical trials and will, therefore, facilitate important clinical trials on asthma in the UK.

## Abbreviations

AUKCAR, Asthma UK Centre for Applied Research; CHI, Community Health Index; DCRU, Duke Clinical Research Unit; EHRs, electronic health records; GP, general practitioner; NHS, National Health Service; PPI, Patient and Public Involvement; RSVP, Research Study Volunteer Programme; SDRN, Scottish Diabetes Research Network; SHARE, Scottish Health Research Register; SQL, Structured Query Language.

## Additional files

Additional file 1: Case studies of success. This is a summary of the key features of existing analogous platforms. (DOCX 24 kb) Additional file 2: Information collected when enrolling into the platform. This contains the questionnaire used for enrolment into the database. (DOCX 22 kb)
