# Supplementary material for: Building a recruitment database for asthma trials: a conceptual framework for the creation of the UK Database of Asthma Research Volunteers
Source: Trials. 2016 May 26;17:264. doi: 10.1186/s13063-016-1381-6 (PMC4882788; doi:10.1186/s13063-016-1381-6)
Supplement: Additional file 1: — Case studies of success. This is a summary of the key features of existing analogous platforms. (DOCX 24 kb) [file 13063_2016_1381_MOESM1_ESM.docx]

## Supplementary File 1:

## Case Studies of Success

In this section we summarise the key features of existing analogous platforms.

*SHARE*

The SHARE platform (www.goshare.org.uk) is a NHS Scotland-funded recruitment infrastructure in Scotland, which aims to recruit one million Scottish residents who are willing to participate in clinical research. SHARE was launched in 2011 and involves a partnership between the Scottish Government and Universities in Scotland. It is a research database which currently consists of people aged 16 years and over who have consented to be informed about clinical research projects that they may be interested in participating. Plans are also in progress to recruit children (<16 years) into the platform. An auxiliary platform (GoSHARE) has been started within SHARE, which consents those registering in SHARE to permit the use of left-over blood samples to be stored under the auspices of a tissue bank and used for future clinical research.

*Scottish Diabetes Research Network*

The Scottish Diabetes Research Network (www.sdrn.org.uk) was created in 2006 to improve the quality and increase the quantity of diabetes research in Scotland; sharing a vision with the UK Clinical Research Collaboration (UKCRC). With core interests in supporting clinical studies, the Scottish Diabetes Research Network fosters collaborations for investigator-led multicentre studies and works with individuals with diabetes in Scotland to enhance participation in high quality clinical studies. The network also conducts nationwide epidemiological analyses of fully anonymised linked datasets and generates research outputs to inform policy. As part of its recruitment and retention strategy, the Scottish Diabetes Research Network has a simple registration procedure and maintains an active online blog and other information sources for its key stakeholders.

*Duke Clinical Research Unit (DCRU)*

The DCRU ([www.dcru.org](http://www.dcru.org)) is a research unit within the Duke Clinical Research Institute, Duke University, which provides the infrastructure to support researchers in the design, initiation, and conduct of clinical research. Part of its core work focuses on recruitment of potential participants to clinical research studies of any design. The unit evaluates recruitment feasibility for each study protocol, sets up realistic recruitment targets, develops recruitment campaign plans, undertakes and manages the recruitment and participant retention during the course of the study. The unit owns the Research Participant Recruitment Core (RPRC) platform, which is a continuously-populated register of research volunteers who have consented to be contacted for future clinical research studies. The RPRC is the first source of recruitment of potential participants into clinical studies. On receiving recruitment requests from investigators, the EHRs of registrants in the RPRC are queried to ascertain a pool of potential participants for specific studies. Additionally, the unit provides different community recruitment strategies before or during the study, including making phone calls and using different print and electronic media to solicit participation into studies. Like the SHARE platform, the RPRC register is a generalist database, but collects information on diagnosed health or medical condition of registrants at the time of enrolment or during screening for trial recruitment.

*Research Study Volunteer Program*

The Research Study Volunteer Program ([www.rsvpforhealth.org](http://www.rsvpforhealth.org)) is a clinical research volunteer register established by the Brigham and Women’s Hospital (BWH) and the Massachusetts General Hospital (MGH), Harvard Medical School, which recruits both healthy and individuals diagnosed with medical conditions of all age groups who are interested in participating in clinical research across every therapeutic area on an ongoing basis. Interested individuals are invited to register their contact details and the therapeutic areas they wish to participate in clinical research. At registration, consent is taken from registrants, with the opportunity of opting out of the database at any time they wish. Registrants’ information is then available to investigators who assess their eligibility to participate in a given study. Information is then sent to eligible participants on any Institution Review Board-approved study taking part in BWH and MGH and that fall within participants’ therapeutic area of interest. Where necessary, participants are compensated for participation in any study.
