# Supplementary material for: Building a recruitment database for asthma trials: a conceptual framework for the creation of the UK Database of Asthma Research Volunteers
Source: Trials. 2016 May 26;17:264. doi: 10.1186/s13063-016-1381-6 (PMC4882788; doi:10.1186/s13063-016-1381-6)
Supplement: Additional file 2: — Information collected when enrolling into the platform. This contains the questionnaire used for enrolment into the database. (DOCX 22 kb) [file 13063_2016_1381_MOESM2_ESM.docx]

# Building a recruitment database for asthma trials: a conceptual framework for the creation of the UK Database of Asthma Research Volunteers

Bright I Nwaru^1,2^, Ireneous N Soyiri^1^, Colin R Simpson^1^, Chris Griffiths^3^, Aziz Sheikh^1^

^1^Asthma UK Centre for Applied Research, Centre for Medical Informatics, Usher Institute of Population Health Sciences and Informatics, The University of Edinburgh, UK

^2^School of Health Sciences, University of Tampere, Finland

^3^Centre for Primary Care and Public Health, Blizard Institute, Queen Mary University of London, UK

Correspondence:

Aziz Sheikh

Asthma UK Centre for Applied Research

Centre for Medical Informatics

Usher Institute of Population Health Sciences and Informatics

The University of Edinburgh, UK

Tel: +44 131 651 4151

Fax: +44 131 650 9119

# Email: aziz.sheikh@ed.ac.uk

# Supplementary File 2: Information collected when enrolling into the platform

### Personal Details

### Name: First name_____________ Last name_______________ Middle initial__

### Date of birth: Day_____ Month_____ Year____

### Gender:

### Smoking status: Currently smoke___ Stopped smoking___ Never smoked___

### Contact details

### Address look up:

### Postcode look up for GP practice:

### Phone

### Email

### Preferred contact method

### How did you hear about us?:

### Asthma status

### Have you ever had wheezing or whistling in the chest at any time, either now or in the past? No/Yes

### Have you had this wheezing or whistling in the chest in the last 12 months? No/Yes

### Did a doctor or nurse ever tell you that you had asthma at any time, either now or in the past? No/Yes

### Have you had any symptoms of asthma in the last 12 months? No/Yes

### Have you been prescribed or received any medications for your asthma/wheezing/whistling in the chest in the last 12 months? No/Yes

### If Yes, was the medication:

### An inhaler?

### A steroid tablet?

### Have you been hospitalised in the last 12 months because of your asthma (including any emergencies)? No/Yes

- Have you had difficulty sleeping because of your asthma symptoms (including cough)? No/Yes
- Have you had your usual asthma symptoms during the day (like cough, wheeze, chest tightness or breathlessness)? No/Yes
- Has your asthma interfered with your usual activities (e.g. housework, work/school, etc)? No/Yes

### Confidentiality

### Any information we collect from you will be kept confidential and stored in a highly secured server at Asthma UK Centre for Applied Research (AUKCAR). Only authorised AUKCAR staff.

### A steering group has been set up within AUKCAR whose mandate is to monitor the operation of the database to ensure that the UK safety and privacy regulations are conformed to.

### Your participation in this database is entirely voluntary and the information you provide will be kept indefinitely. If you decide to withdraw, which you can do at any time, your information will be removed from the database.

### If you decide to participate and register with the database, your information will be made available to approved researchers. These researchers will be able to contact you to participate in AUKCAR approved clinical research studies. AUKCAR staff can also subsequently contact you to follow-up on any future study you decide to participate in.

### In due course, our aim is to be able to link relevant information provided in this database to participant’s GP health records and other routinely collected social, economic, and genetic data sets in the UK. Doing this will then allow us to undertake a more robust and comprehensive investigations into the potential causative and preventive factors for asthma.

### Consent

### To participate, we therefore request that you consent to at least one the following statements. That AUKCAR can:

### register you into the database and link your data to both GP health records and other routinely collected social, economic, genetic data sets in due course

### register you into the database and link your data to only GP health records and other routinely collected data sets

### register you into the database without linking your data to GP health records or other routinely collected data sets
